# Comment on Yorulmaz et al. Enhancing the Prediction of Inborn Errors of Immunity: Integrating Jeffrey Modell Foundation Criteria with Clinical Variables Using Machine Learning. *Children* 2025, *12*, 1259

**DOI:** 10.3390/children13040476

**Published:** 2026-03-30

**Authors:** Kazim Okan Dolu

**Affiliations:** Department of Pediatric Allergy and Immunology, Kanuni Sultan Süleyman Training and Research Hospital, Istanbul 34307, Türkiye; okandolu@gmail.com

I read with great interest the article by Yorulmaz et al. [1], which integrates Jeffrey Modell Foundation (JMF) criteria with clinical variables to predict Inborn Errors of Immunity (IEI). While the study aims to facilitate early detection and timely referral of IEI patients, I would like to raise three points regarding data consistency, feature selection, and potential spectrum bias.

## 1. Data Inconsistency in Table 2

I observed an arithmetic inconsistency in Table 2 of the original article [1]. In the “Total JMF Points” distribution:

- Row “3 points”: Overall is reported as 21, but IEI (19) + Non-IEI (1) sums to 20.
- Row “4 points”: Overall is reported as 20, but IEI (21) + Non-IEI (0) sums to 21.

This internal inconsistency in the descriptive statistics warrants correction to ensure data accuracy and integrity. I kindly ask the authors or editorial office to verify these counts and issue a correction if confirmed.

## 2. Look-Ahead Bias in Feature Selection

A further concern relates to look-ahead bias (or temporal leakage) in feature selection. The study’s SHAP analysis identifies “ICU admission” and “duration of hospitalization” as top predictors [1]. Although the authors acknowledge that “some features may be unavailable at the time of decision-making” [1], including late-stage severity markers like ICU admission in a model intended for front-line clinical triage creates a disconnect between the clinical goal and the model’s design. A child admitted to the ICU typically bypasses the need for an “early warning” score. The heavy reliance on such late-stage features may artificially inflate performance metrics. It would be valuable if the authors could provide a sensitivity (ablation) analysis showing the model’s performance when these late-stage variables are excluded, to demonstrate its true utility in early clinical encounters.

## 3. Potential Spectrum Bias and Generalizability Concerns

The reported performance metrics (AUC: 0.99, Sensitivity: 0.97) are exceptionally high for a single-center study without external validation [1] and may reflect spectrum bias. The non-IEI group had a mean JMF score of 0.34 compared to 3.37 in the IEI group, with ICU admission rates of 3% versus 43.88% [1]. These marked differences suggest the control group may not adequately represent the challenging differential diagnoses encountered in routine clinical practice, potentially limiting the model’s generalizability to real-world populations where the diagnostic distinction is less clear.

In conclusion, while the effort to improve IEI screening via machine learning is commendable, clarifying these issues—particularly through ablation analysis excluding late-stage markers and future validation in diagnostically heterogeneous cohorts—would strengthen the clinical utility and interpretability of this promising work.
